# Retraction: PU.1-deficient mice are resistant to thioacetamide-induced hepatic fibrosis: PU.1 finely regulates Sirt1 expression via transcriptional promotion of miR-34a/-29c in HSCs

**DOI:** 10.1042/BSR20170926_RET

**Published:** 2026-05-06

**Authors:** Qing Liu, Yongming Zhang, Songzhu Yang, Yanfang Wu, Jiantao Wang, Weiwei Yu, Yanguo Liu

**Affiliations:** Department of Hepatobiliary Surgery, Yantaishan Hospital, Yantai, China

**Keywords:** hepatic fibrosis, hepatic stellate cell activation, miR-34a/-29c, PU.1, sirtuins

This article is being retracted from *Bioscience Reports* at the request of the Editor-in-Chief and the Editorial Board. This follows the receipt of a notification from a reader, alerting the Editorial Board to a region of high similarity within Figure 6A of this article. The Editorial Office identified further similarities within the western blot data. Specifically, similarities appear to be present in the following images:

- between regions of the Figure 6A Control and PU1+/- Week 0 panels
- between the Figure 1C PU.1 bands and Figure 2B PU.1 bands
- between the Figure 2D PU.1 bands (Vector and PU.1, and PU.1 and Sirt1 after horizontal flip)
- between the Figure 2D PU.1/Vector band and the Figure 3C PU.1/Vector band
- between the Figure 3C Pu.1 third and fourth bands
- between the Figure 3C Sirt1 third and fourth bands
- between the Figure 4C a-SMA fourth and fifth bands.

The authors responded when contacted regarding the concerns but were unable to provide raw data or explain the similarities. Given the extent of the issues raised, the Editorial Board stands by the decision to retract the article. The authors agree to the retraction.

